# Supplementary material for: S. cerevisiae Cells Can Grow without the Pds5 Cohesin Subunit
Source: mBio. 2022 Jun 16;13(4):e01420-22. doi: 10.1128/mbio.01420-22 (PMC9426526; doi:10.1128/mbio.01420-22)
Supplement: FIG S1 [file mbio.01420-22-s0001.pdf]

A

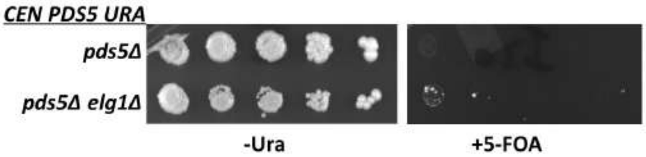

B

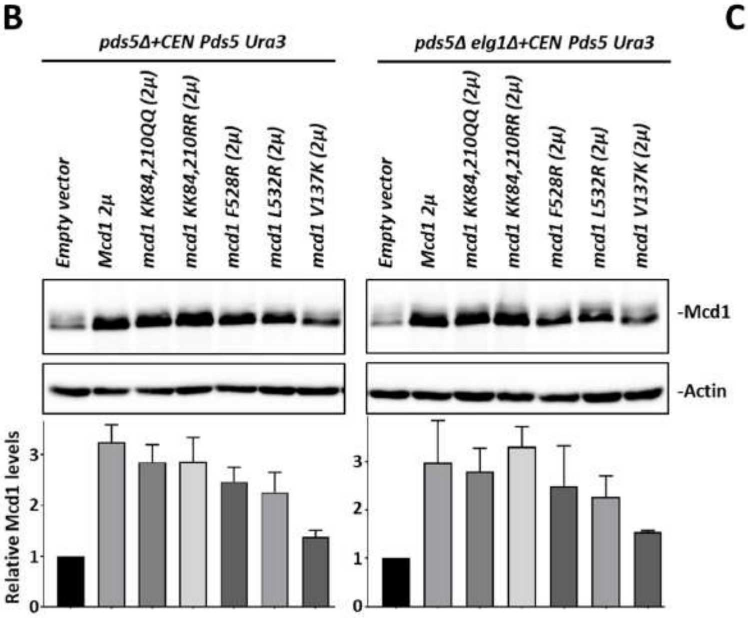

C

GENE NAME	Mutation	No. of times mutation appeared
CLN2	Ser 315 (* stop codon)	2x
CLN2	Arg 100 Ile	2x
CLN2	Gly132 fs (frame shift)	4x
CLN2	Ile186 fs (frame shift)	2x
CLN2	Lys 225 fs (frame shift)	2x
CLN2	Asp 273 (gene deletion)	3x
CLN2	Ile 94 leu, His 97 fs (frame shift)	1x
CLN2	Arg 9 fs (frame shift)	2x
CLN2	Gly 132 fs (frame shift)	3x
CLN2	Ty insertion	1x
CLN2	Pro 470 fs (frame shift)	1x

Figure S1
