# Supplementary figures and images for: S. cerevisiae Cells Can Grow without the Pds5 Cohesin Subunit
Source: mBio. 2022 Jun 16;13(4):e01420-22. doi: 10.1128/mbio.01420-22 (PMC9426526; doi:10.1128/mbio.01420-22)

**A**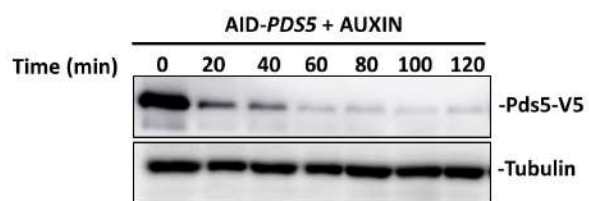**B**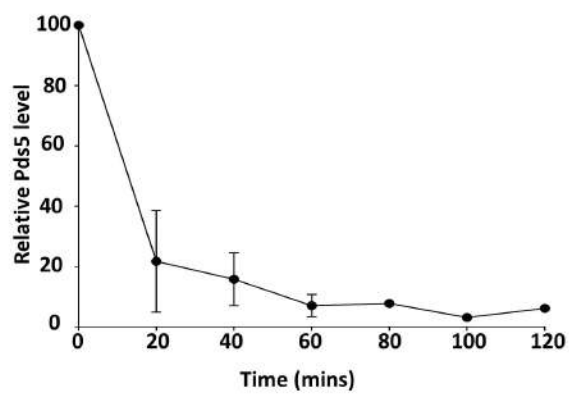**C**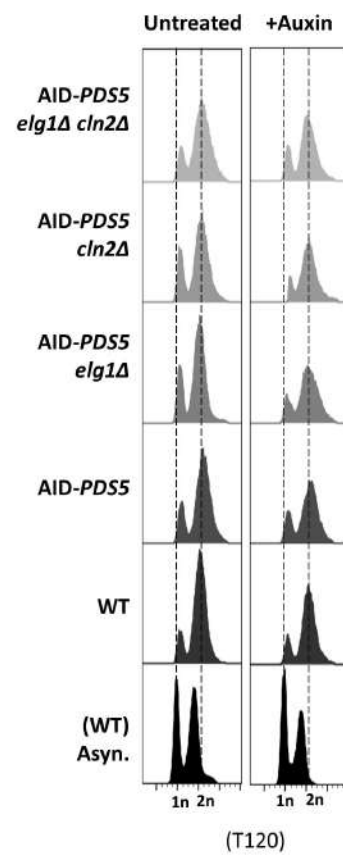

Figure S2

Supplement: FIG S2 [file mbio.01420-22-s0002.pdf]

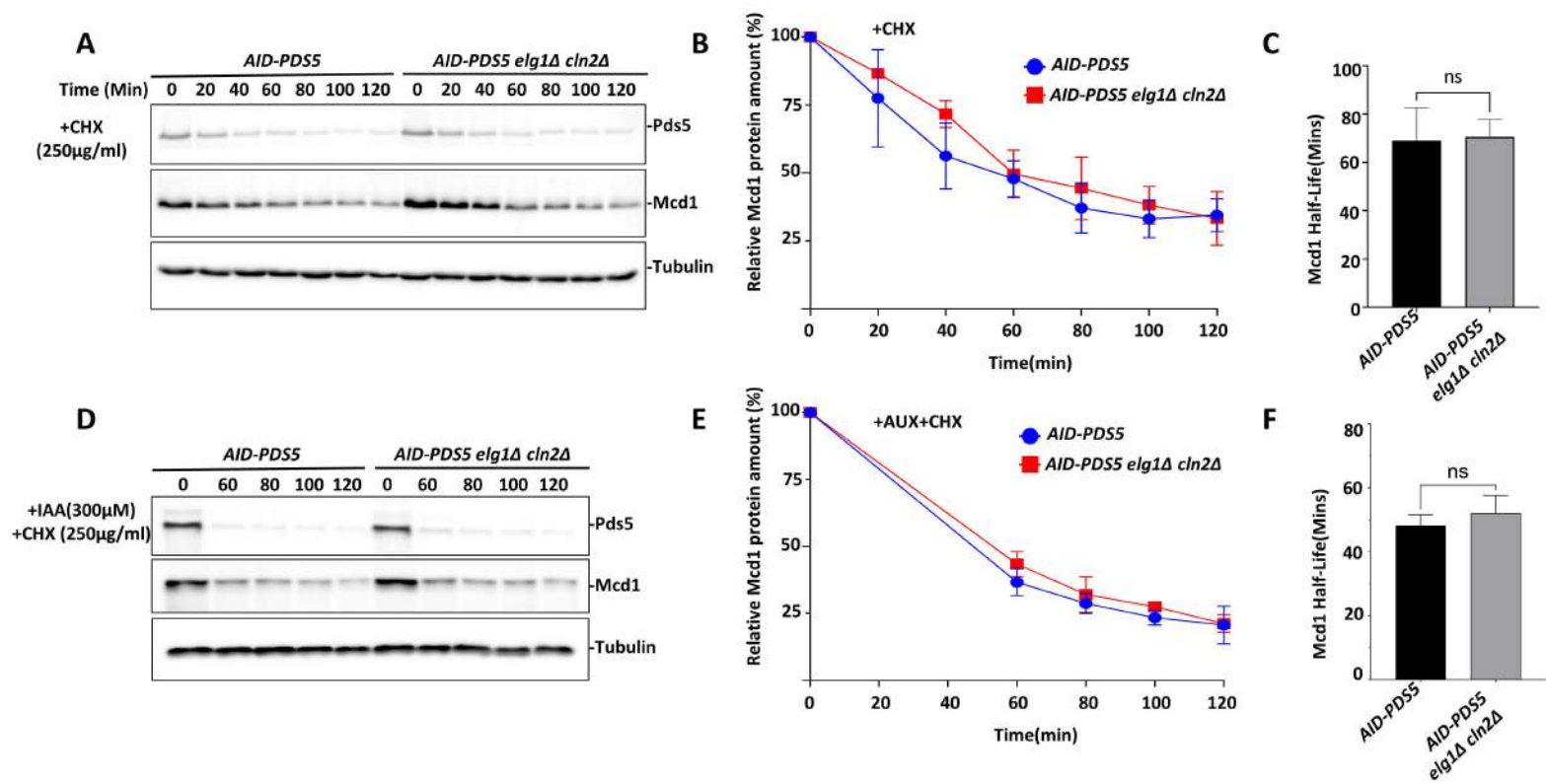

Figure S3

Supplement: FIG S3 [file mbio.01420-22-s0003.pdf]
